# Fabrication of Subretinal 3D Microelectrodes with Hexagonal Arrangement

**DOI:** 10.3390/mi11050467

**Published:** 2020-04-29

**Authors:** Hee Won Seo, Namju Kim, Sohee Kim

**Affiliations:** Department of Robotics Engineering, Daegu Gyeongbuk Institute of Science and Technology (DGIST), Daegu 42988, Korea

**Keywords:** retinal implant, subretinal stimulation, 3D electrodes, hexagonal electrodes, transparent base

## Abstract

This study presents the fabrication of three-dimensional (3D) microelectrodes for subretinal stimulation, to accommodate adjacent return electrodes surrounding a stimulating electrode. For retinal prosthetic devices, the arrangement of return electrodes, the electrode size and spacing should be considered together, to reduce the undesired dissipation of electric currents. Here, we applied the hexagonal arrangement to the microelectrode array for the localized activation of retinal cells and better visual acuity. To provide stimuli more efficiently to non-spiking neurons, a 3D structure was created through a customized pressing process, utilizing the elastic property of the materials used in the fabrication processes. The diameter and pitch of the Pt-coated electrodes were 150 μm and 350 μm, respectively, and the height of the protruded electrodes was around 20 μm. The array consisted of 98 hexagonally arranged electrodes, supported by a flexible and transparent polydimethylsiloxane (PDMS) base, with a thickness of 140 μm. Also, the array was coated with 2 μm-thick parylene-C, except the active electrode sites, for more focused stimulation. Finally, the electrochemical properties of the fabricated microelectrodes were characterized, resulting in the mean impedance of 384.87 kΩ at 1 kHz and the charge storage capacity (CSC) of 2.83 mC·cm^−2^. The fabricated microelectrodes are to be combined with an integrated circuit (IC) for additional in vitro and in vivo experiments.

## 1. Introduction

Retinal degenerative diseases are one of the causes that impair the vision of patients. Age-related macular degeneration (AMD) and retinitis pigmentosa (RP) are the diseases caused by the degeneration of photoreceptors, which finally lead to visual loss [1,2,3,4]. Photoreceptor cells consist of rod and cone cells in the retina, and these cells convert light entering the eye into electrical signals. However, if photoreceptors are lost because of AMD and RP, these signals cannot be generated and transmitted to non-spiking neurons in the inner nuclear layer (INL) [5]. Thus, this results in no responses of the retinal ganglion cells (RGCs) that transmit the signals to the optic nerve.

Hence, to restore the vision in patients with such diseases, various retinal prostheses have been developed, which can replace the function of lost photoreceptors [5,6,7,8,9,10,11,12,13]. The retinal prosthesis, including stimulating electrodes, can partially restore the patient’s vision by stimulating live RGCs using a variety of surgical methods [14]. Depending on the surgical procedure, there are epiretinal electrodes placed on the retinal surface to directly stimulate RGCs [6,11,15,16,17,18], subretinal electrodes implanted between the outer nuclear layer (ONL) and the retinal pigment epithelium (RPE) [7,8,9,10,19,20,21,22,23,24], and suprachoroidal electrodes implanted between the choroid and the sclera [12].

Mostly, the stimulating electrodes have been developed to stimulate retinal cells epiretinally or subretinally [6,7,8,9,10,11], which differ in their stimulation thresholds to electrically elicit the responses of RGCs. The stimulation threshold can vary depending on the type of pulse applied to the electrode (monophasic or biphasic), pulse polarity (cathodic or anodic), and pulse duration [25,26,27,28,29]. Typically, the epiretinal electrodes, to elicit direct RGC responses, have low stimulation thresholds with pulses of shorter durations of around 0.5 ms, whereas the subretinal electrodes, which avoid direct activation of RGCs, have relatively low stimulation thresholds, with pulses of longer durations ranging from 1 to 10 ms [8,25,29]. For subretinal electrodes, stimulating pulses are transmitted to non-spiking neurons in INL (horizontal, bipolar and amacrine cells), and due to the slow nature of these neurons, stimuli with moderately long pulse durations (1 to 10 ms) help reduce the thresholds [5,25].

Previously, we focused on the subretinal approach that can deliver sufficient stimulation to the cells in the INL, and demonstrated a three-dimensional subretinal electrode array, based on a flexible and transparent base [30], which, however, had a small number of electrodes; up to 25 in an area of 2.8 mm × 2.8 mm. Here, we present a subretinal electrode array with an increased number of electrodes to anticipate better visual acuity. For that, a large number of electrodes need to be accommodated within the limited size of the device. In addition, when an electric field is generated by the stimulating electrodes, the arrangement of return electrodes, the electrode size and spacing should be considered together to reduce undesired dissipation of electric currents, i.e., crosstalk [31,32]. Based on such previous results, we aimed to implement the hexagonal return electrodes surrounding a stimulating electrode in center, to achieve the localized activation of RGCs on stimulating sites. Moreover, by adapting the hexagonal electrode arrangement, advantages such as more correct identification of symbols [33] and a higher electrode density within the limited device area [34] can be anticipated. Finally, we fabricated a three-dimensional (3D) microelectrode array with 98 electrodes based on the transparent base and characterized the electrochemical properties of the fabricated electrodes, prior to in vitro and in vivo experiments.

## 2. Materials and Methods

### 2.1. Design of Subretinal Electrode Array

**Figure 1:**
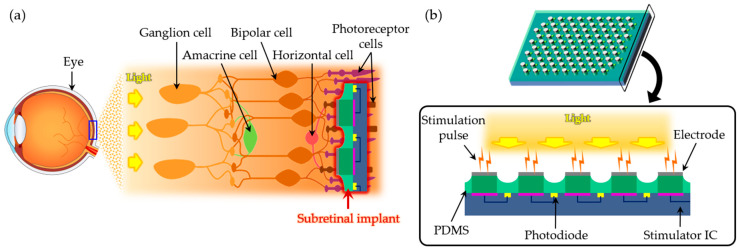
Schematic illustrations of microelectrodes for subretinal stimulation. (**a**) The subretinal implant is placed between the outer nuclear layer (ONL) and the retinal pigment epithelium (RPE) to stimulate non-spiking neurons. (**b**) Stimulation pulses are generated when photodiodes in the implant convert light into electrical signals.

Figure 1 shows the schematic illustrations of the 3D microelectrodes for subretinal stimulation. The microelectrodes, which can be combined with an integrated circuit (IC), are subretinally implantable, as shown in Figure 1a. The electrode array consists of 98 hexagonally arranged electrodes, and the sensed light can be converted into the electrical signal by the photodiodes in the IC that is to be integrated with the electrodes. For the photodiodes to detect light directly, polydimethylsiloxane (PDMS) was used as the transparent base of the electrodes, as shown in Figure 1b. The thickness of PDMS was chosen to be 140 μm. The distance between the centers of two neighboring electrodes was set to be 350 μm, and the height of the 3D electrodes was set to be around 20 μm.

### 2.2. Generation of Silicon Pillars and Transparent Base

**Figure 2:**
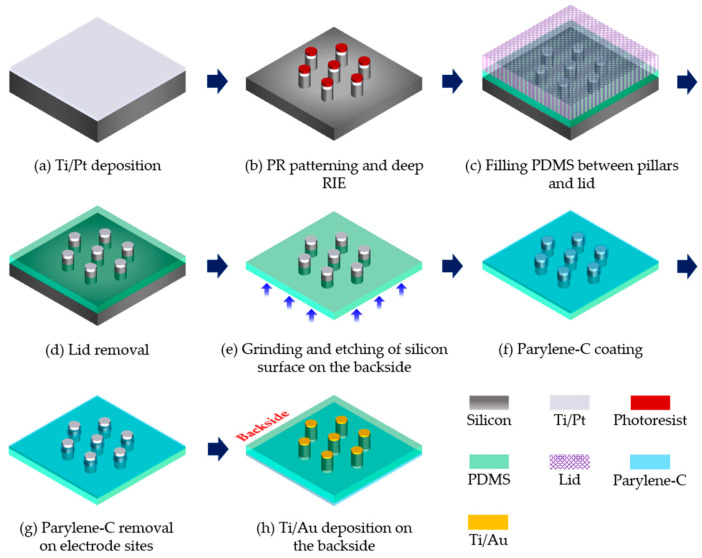
Schematic diagram of the main processes to fabricate the 3D microelectrodes in a hexagonal arrangement.

A p-type silicon wafer was used to fabricate the 3D microelectrodes. The diameter and thickness of the used silicon wafer were 6-inch and 675 μm, respectively. First, Ti and Pt were deposited on the silicon wafer using a sputtering process (SRN-110, Sorona, Anseong, Korea), which served as the metal layer of the electrodes, as shown in Figure 2a. Ti and Pt layers were deposited with thicknesses of 50 nm and 200 nm, respectively, and patterned using a photoresist (AZ5214, MicroChemicals, Ulm, Germany).

**Figure 3:**
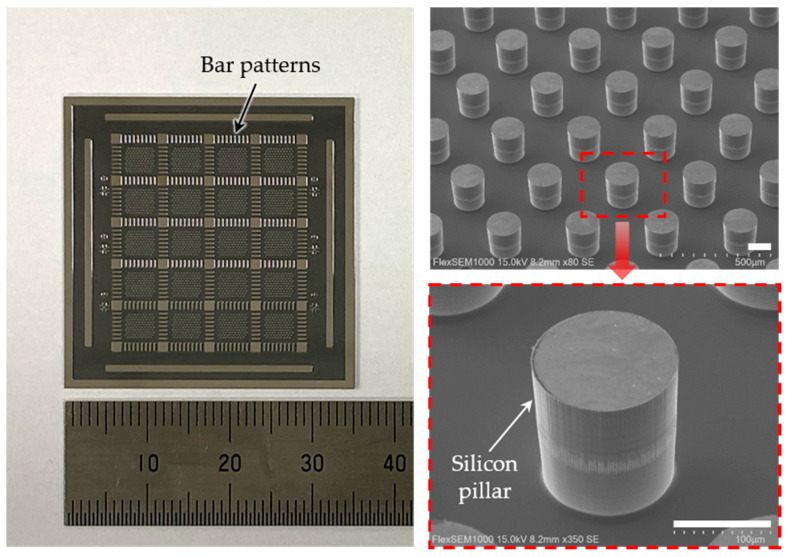
(**Left**) Photograph of a chip containing 20 arrays of circular silicon pillars, in a dimension of 35 mm × 35 mm. (**Right**) Scanning electron microscopic (SEM) images of silicon pillars after deep reactive ion etching. The scale bars are 100 μm.

After patterning the metal layer, 3D pillars were created in the silicon wafer by using a deep reactive ion etching (DRIE) process (LPX PEGASUS, SPTS, Allentown, PA, USA), along with the designed electrode patterns, as shown in Figure 2b. An 8 μm-thick photoresist mask (AZ9260, MicroChemicals, Ulm, Germany) was used for DRIE. The height of the silicon pillars was chosen to be 160 μm. In order to easily fill the transparent PDMS between the fabricated silicon pillars, the silicon wafer was cut into nine chips using a dicing process (DAD3240, Disco, Tokyo, Japan). A chip contained 20 electrode arrays in a dimension of 35 mm × 35 mm, as shown in Figure 3. In the chip, the bar patterns surrounding the electrodes were also created to guide the cutting of the chip into the electrode arrays for the final step.

Next, to create the transparent and flexible base, PDMS (Sylgard^™^ 184, Dow Corning, Midland, MI, USA) was filled between the silicon pillars. After oxygen plasma (CUTE-B, Femtoscience, Hwaseong, Korea) was treated on the chip to enhance the adhesion between the PDMS to be filled and the chip surface, liquid PDMS was filled into the space generated between the pillars and the ‘lid’, as shown in Figure 2c. The PDMS was mixed with curing agent at a 10:1 weight ratio. The lid, which covered the top of the silicon pillars, was made of the same PDMS mixture coated with 3 μm-thick parylene-C. In this process, the elastic PDMS lid was uniformly pressured using binder clips to realize a 3D electrode shape. A slide glass was used on the lid for the uniform distribution of pressure by the binder clips, and the lid evenly covered the top of the silicon pillars. After the PDMS was filled into the space between the chip and the lid, microbubbles contained in the liquid PDMS were removed using a vacuum desiccator (SW-V200D, Samwoo Engineering, Seoul, Korea). Then, the chip was cured in an oven at 60 °C for 4 hours, and separated from the lid, as shown in Figure 2d. As a result, a 3D structure by the silicon electrodes in combination with the PDMS base was created by the pressure applied to the elastic lid.

### 2.3. Generation of Connection Pads and Parylene Insulation

To completely reveal the PDMS on the opposite side of the chip, the silicon on the backside was removed. First, the bulk of the silicon was removed physically through a grinding process (DA08AG, KTL, Anseong, Korea), leaving only 50 μm-thick silicon. An isotropic wet etching process was performed to etch the remaining silicon, where a mixture of HF (48%) and HNO_3_ (69%) in a 1:3 volume ratio was used [35]. The solution was agitated at 500 rpm for 10 minutes using a magnet stirrer (WiseStir^®^ MSH-20D, DAIHAN Scientific, Wonju, Korea). After the chip was fixed to a custom-designed jig, it was immersed in the solution. As the first step, the silicon surface was etched until the PDMS began to appear, and the etching time was about 7 minutes, with no agitation of the solution. Next, the mixed solution of HF and HNO_3_ with a 1:5 volume ratio was used for the second etching step. The solution was changed to create the pads with a relatively slow chemical reaction, and the wet etching was performed until the height of the pads reached the PDMS base. In this step, the etching time was about 4 minutes, with no agitation of the solution. To remove the residual solution, the chip was washed with deionized water for 2 minute and baked on a hotplate at 110 °C for 10 minutes [30]. The transparent base of the electrodes was completely exposed, as shown in Figure 2e.

Next, an insulating layer was coated on the topside of the microelectrode array for more focused stimulation. As shown in Figure 2f, 2 μm-thick parylene-C was coated (NRPC-500, Nuritech, Incheon, Korea). An adhesion promoter (Silquest A-174, Merck, Darmstadt, Germany) was used to enhance the adhesion between parylene-C and the topside of the microelectrodes. Then, a dry etching process was performed to expose the electrode sites, using a patterned mask made of a photoresist (AZ9260, MicroChemicals, Ulm, Germany), with a thickness of 11 μm. Using a reactive ion etching process (VITA, Femtoscience, Hwaseong, Korea), parylene-C on the electrode sites was removed with a power of 200 W and an oxygen gas flow of 100 sccm for 15 minutes, as shown in Figure 2g.

To create the connection pads for the electrodes, metal layers were deposited on the backside of the electrodes, so that the microelectrodes could be electrically connected with the IC. First, the chip was cut into individual electrode arrays using a cutter (DN-52, Dorco, Seoul, Korea) for an accurate sputtering process. To deposit the metal layers only on the etched silicon surface, not on the PDMS surface, the sputtering process was performed using a patterned mask. The used mask was a 40 μm-thick polyethylene terephthalate (PET) film patterned by a UV laser machine (ALM100, Sejoong Information Technology, Seoul, Korea). The patterned mask was manually aligned on the backside of the electrodes under a microscope (SZMN45TR-STL1, Sunny Optical Technology, Yuyao, China). Using a sputtering process (DKC161125, Daeki Hi-Tech, Daejeon, Korea), Ti and Au layers were deposited with thicknesses of 50 nm and 200 nm, respectively, on the silicon surface. Figure 2h shows the connection pads created on the opposite side of the electrodes.

### 2.4. Electrochemical Characterization

Electrochemical impedance spectroscopy (EIS) and cyclic voltammetry (CV) were performed to investigate the electrical properties of the fabricated microelectrodes. The electrodes were connected using a custom-designed parylene-C cable fabricated based on a previous study [36]. To connect the pads of the electrode array with the parylene-C cable, conductive epoxy (Duralco^™^ 125-2, Contronics, Brooklyn, NY, USA) was used, and the assembled device was cured in an oven at 35 °C for 16 hours. Next, the parylene-C cable was connected to a printed circuit board (PCB), through a zero-insertion-force (ZIF) connector. As the ZIF connector (502598-3393, Molex, Lisle, IL, USA) had only 33 channels, all 98 electrodes could not be connected to the connector at the same time. Thus, we designed the parylene-C cable to have three parts of connection pads: the first part had 32 electrical lines and pads to connect 32 electrodes in the center of the array, while the second and third parts had 33 electrical lines and pads each, to connect the remaining electrodes. For EIS, a potentiostat (Reference 600+, Gamry Instruments, Warminster, PA, USA) and a custom-designed faraday cage with a dimension of 30 cm × 38 cm × 52 cm were used. The fabricated electrode array, an Ag/AgCl electrode, and a platinum wire were used as the working electrodes, the reference electrode, and the counter electrode, respectively. The prepared electrodes were immersed in phosphate buffer saline (PBS) solution (PBS 10010023, Thermo Fisher Scientific, Waltham, MA, USA), and the frequency was swept from 10 Hz to 1 MHz with a sinusoidal AC input voltage of 30 mV_rms_. For CV, the potential limits were set from −0.6 V to 0.8 V, at a scan rate of 50 mV·s^−1^ [30,37].

## 3. Results and Discussion

**Figure 4:**
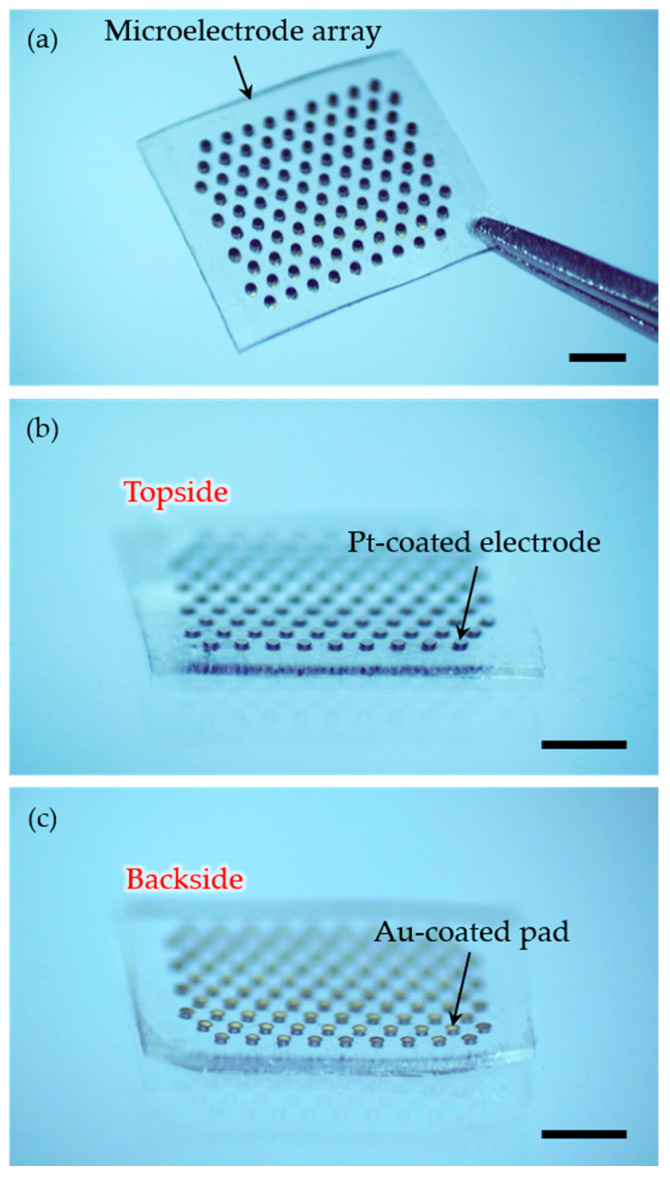
Photographs of the fabricated 3D microelectrode array: (**a**) top view of the array in a dimension of 4.4 mm × 3.8 mm and (**b**,**c**) its side views showing (**b**) the topside containing stimulating electrode sites and (**c**) the backside containing the connection pads. The scale bars are 1 mm.

The 3D microelectrode array with 98 hexagonally arranged electrodes was fabricated using micro-electro-mechanical systems (MEMS) technologies. Figure 4 shows the photographs of the completed 3D microelectrodes. 98 electrodes were arranged in a dimension of 4.4 mm × 3.8 mm within an array, and the outermost electrodes were designed to be used as return electrodes only. If an electrode is selected as the stimulating electrode except for the outermost electrodes, six surrounding return electrodes can guard the stimulating electrode, resulting in 64 effective pixels in the vision. The completed electrodes had the diameter and pitch of 150 μm and 350 μm, respectively, supported by a 140 μm-thick PDMS base, and the topside of the array, except the electrode sites, was coated with 2 μm-thick parylene-C.

**Figure 5:**
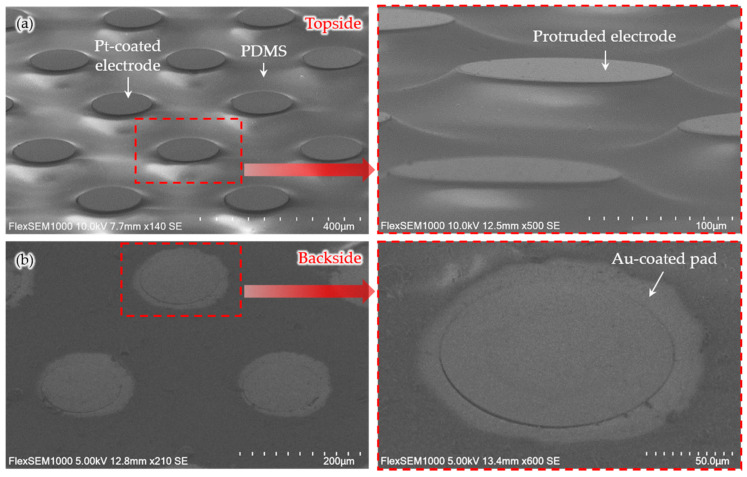
SEM images of the microelectrodes. (**a**) Three-dimensionally protruded electrodes created by the pressing process using an elastic lid. (**b**) Au-coated connection pads on the opposite side of the electrodes.

Figure 5 shows the scanning electron microscopic (SEM) images (FlexSEM 1000, Hitachi, Tokyo, Japan) of the microelectrodes. The Pt-coated electrodes protruded around 20 μm from the PDMS base, as shown in Figure 5a. The height of the protruded electrodes can be controlled by both the pressing strength of the binder clips and the strength of the PDMS lid. The higher the pressure of the binder clips, the stronger the elastic PDMS lid can press the PDMS between the silicon pillars, resulting in more protruded electrodes. On the other hand, if the pressure of the binder clips is constant, the height of the protruded electrodes can be varied according to the elasticity of the lid itself. The elasticity of the lid may depend on the mixing ratio, curing temperature, and curing time [38,39]. Figure 5b shows the Au-coated connection pads on the backside of the microelectrodes. Although the diameter of the patterned mask was set to be the same as the diameter of the pads, the metal layers were deposited slightly wider than the pad area. Since the mask physically covered the pads, resulting in a little gap, metal ions spread beyond the pad area. However, it can be easily corrected by reducing the diameter of the masking pattern.

**Figure 6:**
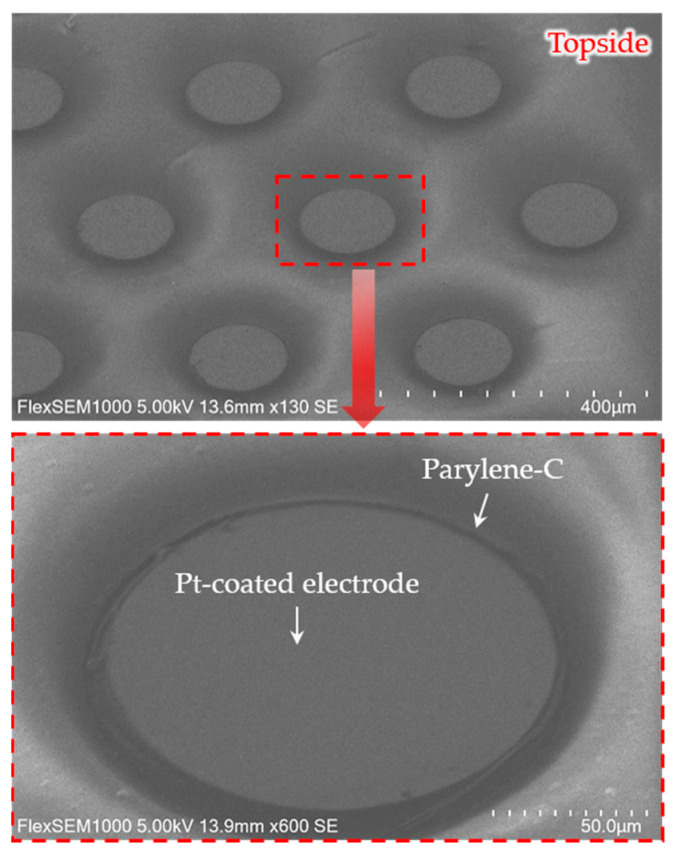
SEM images of the electrode array insulated by parylene-C, excluding the electrode sites.

Figure 6 shows the parylene-C-coated microelectrode array with the exposed electrode sites, where parylene-C was removed. As shown in Figure 6, parylene-C residue on the electrode site was completely removed. Parylene has been suggested as a suitable coating material due to the great barrier property [40,41]. Previously reported studies investigated the biocompatibility of parylene implanted in the retina [42,43]. In [43], the biocompatibility of a parylene-based retinal device implanted subretinally was investigated for three months, in which reactions such as fibrosis and tissue damage did not occur around the implant. However, as an encapsulation material, the very long-term in vivo performance of parylene needs to be evaluated for chronic implants.

In our previous work [30], we fabricated 3D electrodes with a grid arrangement in an area of 2.8 mm × 2.8 mm, based on the dicing of bulk silicon. Such physical removal of silicon, however, has limitations in fabricating the array with an increased number of electrodes within the limited device area, and in that electrode arrangement other than grid or rectangular arrangement is not possible. On the contrary, the current study suggests that the new fabrication method can overcome such limitations. Through the chemical etching of silicon, it was possible to accommodate more electrodes with smaller sizes within the same area of the device, particularly in any arrangement, such as grid, rectangular, hexagonal, and octagonal arrangements. We successfully realized 98 hexagonally arranged electrodes in an area of 4.4 mm × 3.8 mm using the proposed fabrication method, while only 40 electrodes could have been constructed if they were arranged in a grid and fabricated using the previous method published in [30]. Nevertheless, compared to the previous studies by others [7,44], the number of our fabricated electrodes is relatively insufficient, although we have increased the number of electrodes from 25 in our previous study to 98 electrodes in the current study. In [7], silicon-based electrode arrays with 250 pixels were fabricated, which had a height of 10 μm and a diameter of 14 μm. To achieve better visual acuity, our array should be improved with a much increased number of electrodes within the limited device area, such as those reported in the previous studies [7,22,44]. Using the proposed fabrication method, we anticipate that it would be possible to decrease the electrode size down to 70 μm, increasing the number of electrodes up to 260 within the same area of the device.

The hexagonal electrode arrangement allows higher electrode densities compared to the rectangular arrangement [34]. Thus, the hexagonal arrangement not only makes it possible to allow a higher resolution of the phosphene image in the same area allowed for a device, but would also have slight advantages in acuity tasks [33,34]. A simulation study reported that by using guarded hexagonal return electrodes, effective retinal stimulation can be ensured, if the diameter of the electrodes under 100 μm is used and the distance between the electrodes and the target neurons is far enough [31]. The effectiveness of the hexagonal return electrodes in minimizing crosstalk between pixels and localized activation of retinal cells, however, needs to be investigated in in vitro as well as in vivo environments, using the fabricated electrodes.

**Figure 7:**
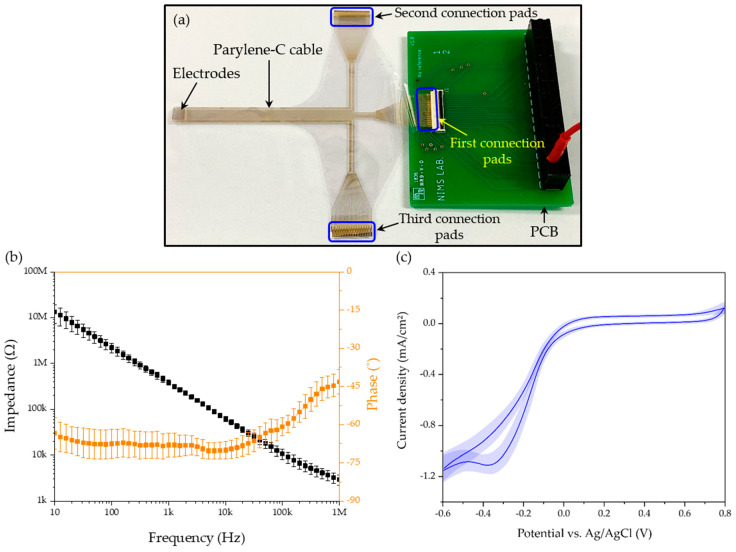
(**a**) Completed electrode array integrated with parylene-C cable and printed circuit board (PCB), for electrochemical characterization. (**b**) Measured impedance of fabricated microelectrodes. Means and standard deviations for magnitude and phase are shown. (**c**) Cyclic voltammogram curves of the Pt-coated electrodes measured at a scan rate of 50 mV·s^−1^.

Figure 7 shows the assembled device for electrochemical characterization and the results. The electrode array combined with the parylene-C cable was connected to a printed circuit board (PCB) for the impedance and CV measurement, as shown in Figure 7a. Overall, 32 microelectrodes were connected to the ZIF connector through the first connection pads of the parylene-C cable. The second and third parts of connection pads were not used in the initial electrode characterization, but reserved for the next experiments using all electrodes in the near future. The impedance of the microelectrodes was measured to be 384.87 ± 52.11 kΩ at 1 kHz (Figure 7b). In the array, the area of an exposed electrode was about 0.018 mm^2^, and the measured impedance at 1 kHz was 21.4 MΩ·mm^−2^ when converted to a value per unit area. In the previous studies [30,37], the unit-area impedances of Pt-coated 3D electrodes were 30.3 MΩ·mm^−2^ and 62.5 MΩ·mm^−2^, respectively. The obtained impedance using the fabricated electrodes was comparable to those values. The CV of the electrodes was also measured, as shown in Figure 7c. The calculated CSC was 2.83 ± 0.22 mC·cm^−2^ and comparable to the values reported in the previous studies [30,37], ranging from 1.24 mC·cm^−2^ to 4.4 mC·cm^−2^.

Although Pt-coated electrodes with impedances in such a range were demonstrated to be able to stimulate the retinal cells [30], electrode materials capable of injecting higher charge are desirable to elicit RGC responses securely and to minimize tissue damage in the stimulated area. Typically, the electrode materials with low impedance show high charge injection capacity (CIC), and higher CIC can lead to higher current density in safe voltage limits, without undesired electrochemical reactions [37]. For this, it would be better to change the electrode material to the one that can result in lower impedance in the same area. For example, activated iridium oxide film (AIROF) or sputtered iridium oxide film (SIROF) can lower the impedance of the electrodes further down to tens of kΩ [37,45,46]. In previously reported studies [7,11,21,22,47,48], various types of retinal electrodes were fabricated using AIROF or SIROF to achieve the low electrode impedance and high CIC. A previous study investigated the threshold charge density according to electrode sizes [49]. Low threshold charge densities were obtained using a large stimulation field of electrodes (≥ 0.078 mm^2^), coated with SIROF. Assuming the same experimental condition that iridium oxide were used as the electrode material, the fabricated electrodes (with an area of 0.018 mm^2^) were expected to have the threshold charge density of about 50 μC·cm^−2^ based on the results presented in [49]. As further studies, we are planning to change the material of the electrodes and verify the subretinal stimulation of the fabricated electrodes in in vitro and in vivo environments.

## 4. Conclusions

We have developed a hexagonally arranged 3D microelectrode array for subretinal prostheses. The base of the array was made of transparent PDMS, so that the photodiodes of the IC connected to the electrodes can sense light directly. By a customized pressing process utilizing the elastic property of the used material, a 3D structure of the electrode array was created, resulting in the electrodes that protruded around 20 μm from the PDMS base. The height of the protruded electrodes can be controlled by varying the pressing strength of the PDMS filled between the silicon pillars. The hexagonal arrangement of electrodes was realized through the chemical etching of silicon, with which any arrangement of electrodes other than hexagonal arrangement is also possible. Such hexagonally arranged electrodes are expected to stimulate retinal cells with a larger number of electrodes in a limited device area, compared to the rectangular arrangement of electrodes. By considering the pitch and size of the electrodes, the 3D microelectrodes can be expanded to have more pixels for even higher resolution if desired. In the future, in vitro and in vivo experiments will be conducted to evaluate the stimulating performance of the fabricated 3D microelectrodes; in particular, the effectiveness of hexagonal return electrodes.

## References

[1] Hartong D.T., Berson E.L., Dryja T.P. (2006). Retinitis pigmentosa. Lancet.

[2] Kim S.Y., Sadda S., Pearlman J., Humayun M.S., de Juan E., Melia B.M., Green W.R. (2002). Morphometric analysis of the macula in eyes with disciform age-related macular degeneration. Retina.

[3] Bressler N.M. (2004). Age-related macular degeneration is the leading cause of blindness. JAMA.

[4] Friedman D.S., O’Colmain B.J., Muñoz B., Tomany S.C., McCarty C., de Jong P.T., Nemesure B., Mitchell P., Kempen J. (2004). Prevalence of age-related macular degeneration in the united states. Arch. Ophthalmol..

[5] Goetz G.A., Palanker D.V. (2016). Electronic approaches to restoration of sight. Reports Prog. Phys..

[6] Ferlauto L., Airaghi Leccardi M.J.I., Chenais N.A.L., Gilliéron S.C.A., Vagni P., Bevilacqua M., Wolfensberger T.J., Sivula K., Ghezzi D. (2018). Design and validation of a foldable and photovoltaic wide-field epiretinal prosthesis. Nat. Commun..

[7] Ho E., Lei X., Flores T., Lorach H., Huang T., Galambos L., Kamins T., Harris J., Mathieson K., Palanker D. (2019). Characteristics of prosthetic vision in rats with subretinal flat and pillar electrode arrays. J. Neural Eng..

[8] Tong W., Stamp M., Apollo N.V., Ganesan K., Meffin H., Prawer S., Garrett D.J., Ibbotson M.R. (2019). Improved visual acuity using a retinal implant and an optimized stimulation strategy. J. Neural Eng..

[9] Davidsen R.S., Hemanth S., Keller S.S., Bek T., Hansen O. (2019). Evaluation of the capacitive behavior of 3D carbon electrodes for sub-retinal photovoltaic prosthesis. Micro Nano Eng..

[10] Losada P.G., Rousseau L., Grzeskowiak M., Valet M., Nguyen D., Dégardin J., Dubus E., Picaud S., Lissorgues G. (2019). Protuberant electrode structures for subretinal electrical stimulation: Modeling, fabrication and in vivo evaluation. Front. Neurosci..

[11] Lohmann T.K., Haiss F., Schaffrath K., Schnitzler A.C., Waschkowski F., Barz C., Van Der Meer A.M., Werner C., Johnen S., Laube T. (2019). The very large electrode array for retinal stimulation (VLARS)-A concept study. J. Neural Eng..

[12] Bareket L., Barriga-Rivera A., Zapf M.P., Lovell N.H., Suaning G.J. (2017). Progress in artificial vision through suprachoroidal retinal implants. J. Neural Eng..

[13] Zeng Q., Zhao S., Yang H., Zhang Y., Wu T. (2019). Micro/nano technologies for high-density retinal implant. Micromachines.

[14] Gabel V.P. (2017). Artificial Vision.

[15] Luo Y.H.L., da Cruz L. (2016). The Argus^®^ II retinal prosthesis system. Prog. Retin. Eye Res..

[16] Ahuja A.K., Dorn J.D., Caspi A., McMahon M.J., Dagnelie G., DaCruz L., Stanga P., Humayun M.S., Greenberg R.J. (2011). Blind subjects implanted with the Argus II retinal prosthesis are able to improve performance in a spatial-motor task. Br. J. Ophthalmol..

[17] Dorn J.D., Ahuja A.K., Caspi A., Da Cruz L., Dagnelie G., Sahel J.A., Greenberg R.J., McMahon M.J. (2013). The detection of motion by blind subjects with the epiretinal 60-electrode (Argus II) retinal prosthesis. JAMA Ophthalmol..

[18] Humayun M.S., Dorn J.D., Da Cruz L., Dagnelie G., Sahel J.A., Stanga P.E., Cideciyan A.V., Duncan J.L., Eliott D., Filley E. (2012). Interim results from the international trial of second sight’s visual prosthesis. Ophthalmology.

[19] Zrenner E., Bartz-Schmidt K.U., Benav H., Besch D., Bruckmann A., Gabel V.P., Gekeler F., Greppmaier U., Harscher A., Kibbel S. (2011). Subretinal electronic chips allow blind patients to read letters and combine them to words. Proc. R. Soc. B Biol. Sci..

[20] Kelly S.K., Shire D.B., Chen J., Doyle P., Cogan S.F., Gingerich M.D., Drohan W.A., Behan S., Theogarajan L., Wyatt J.L. (2011). A hermetic wireless subretinal neurostimulator for vision prostheses. IEEE Trans. Biomed. Eng..

[21] Wang L., Mathieson K., Kamins T.I., Loudin J.D., Galambos L., Goetz G., Sher A., Mandel Y., Huie P., Lavinsky D. (2012). Photovoltaic retinal prosthesis: Implant fabrication and performance. J. Neural Eng..

[22] Mathieson K., Loudin J., Goetz G., Huie P., Wang L., Kamins T.I., Galambos L., Smith R., Harris J.S., Sher A. (2012). Photovoltaic retinal prosthesis with high pixel density. Nat. Photonics.

[23] Flores T., Lei X., Huang T., Lorach H., Dalal R., Galambos L., Kamins T., Mathieson K., Palanker D. (2018). Optimization of pillar electrodes in subretinal prosthesis for enhanced proximity to target neurons. J. Neural Eng..

[24] Stingl K., Schippert R., Bartz-Schmidt K.U., Besch D., Cottriall C.L., Edwards T.L., Gekeler F., Greppmaier U., Kiel K., Koitschev A. (2017). Interim results of a multicenter trial with the new electronic subretinal implant alpha AMS in 15 patients blind from inherited retinal degenerations. Front. Neurosci..

[25] Boinagrov D., Pangratz-Fuehrer S., Goetz G., Palanker D. (2014). Selectivity of direct and network-mediated stimulation of the retinal ganglion cells with epi-, sub- and intraretinal electrodes. J. Neural Eng..

[26] Jensen R.J., Ziv O.R., Rizzo J.F. (2005). Thresholds for activation of rabbit retinal ganglion cells with relatively large, extracellular microelectrodes. Investig. Ophthalmol. Vis. Sci..

[27] Jensen R.J., Rizzo J.F. (2006). Thresholds for activation of rabbit retinal ganglion cells with a subretinal electrode. Exp. Eye Res..

[28] Jensen R.J., Rizzo J.F. (2009). Activation of ganglion cells in wild-type and rd1 mouse retinas with monophasic and biphasic current pulses. J. Neural Eng..

[29] Tsai D., Morley J.W., Suaning G.J., Lovell N.H. (2009). Direct activation and temporal response properties of rabbit retinal ganglion cells following subretinal stimulation. J. Neurophysiol..

[30] Seo H.W., Kim N., Ahn J., Cha S., Goo Y.S., Kim S. (2019). A 3D flexible microelectrode array for subretinal stimulation. J. Neural Eng..

[31] Wilke R.G.H., Moghadam G.K., Lovell N.H., Suaning G.J., Dokos S. (2011). Electric crosstalk impairs spatial resolution of multi-electrode arrays in retinal implants. J. Neural Eng..

[32] Abramian M., Lovell N.H., Habib A., Morley J.W., Suaning G.J., Dokos S. (2014). Quasi-monopolar electrical stimulation of the retina: A computational modelling study. J. Neural Eng..

[33] Chen S.C., Hallum L.E., Lovell N.H., Suaning G.J. (2005). Visual acuity measurement of prosthetic vision: A virtual-reality simulation study. J. Neural Eng..

[34] Vurro M., Baselli G., Orabona F., Sandini G. (2006). Simulation and assessment of bioinspired visual processing system for epi-retinal prostheses. Annu. Int. Conf. IEEE Eng. Med. Biol. - Proc..

[35] Hamzah A.A., Abd Aziz N., Yeop Majlis B., Yunas J., Dee C.F., Bais B. (2012). Optimization of HNA etching parameters to produce high aspect ratio solid silicon microneedles. J. Micromechan. Microeng..

[36] Kang Y.N., Chou N., Jang J., Byun D., Kang H., Moon D., Kim J., Kim S. (2019). An intrafascicular neural interface with enhanced interconnection for recording of peripheral nerve signals. IEEE Trans. Neural Syst. Rehabil. Eng..

[37] Negi S., Bhandari R., Rieth L., Solzbacher F. (2010). In vitro comparison of sputtered iridium oxide and platinum-coated neural implantable microelectrode arrays. Biomed. Mater..

[38] Hocheng H., Chen C.M., Chou Y.C., Lin C.H. (2010). Study of novel electrical routing and integrated packaging on bio-compatible flexible substrates. Microsyst. Technol..

[39] Wang Z., Volinsky A.A., Gallant N.D. (2014). Crosslinking effect on polydimethylsiloxane elastic modulus measured by custom-built compression instrument. J. Appl. Polym. Sci..

[40] Kim S.J., Lee D.S., Kim I.G., Sohn D.W., Park J.Y., Choi B.K., Kim S.W. (2012). Evaluation of the biocompatibility of a coating material for an implantable bladder volume sensor. Kaohsiung J. Med. Sci..

[41] Xie X.Z., Rieth L., Tathireddy P., Solzbacher F. (2011). Long-term in-vivo investigation of parylene-C as encapsulation material for neural interfaces. Procedia Eng..

[42] Montezuma S.R., Loewenstein J., Scholz C., Rizzo J.F. (2006). Biocompatibility of materials implanted into the subretinal space of Yucatan pigs. Investig. Ophthalmol. Vis. Sci..

[43] Yu W., Wang X., Zhao C., Yang Z., Dai R., Dong F. (2009). Biocompatibility of subretinal parylene-based Ti/Pt microelectrode array in rabbit for further artificial vision studies. J. Ocul. Biol. Dis. Infor..

[44] Lorach H., Goetz G., Smith R., Lei X., Mandel Y., Kamins T., Mathieson K., Huie P., Harris J., Sher A. (2015). Photovoltaic restoration of sight with high visual acuity. Nat. Med..

[45] Cogan S.F., Troyk P.R., Ehrlich J., Gasbarro C.M., Plante T.D. (2007). The influence of electrolyte composition on the in vitro charge-injection limits of activated iridium oxide (AIROF) stimulation electrodes. J. Neural Eng..

[46] Cogan S.F., Ehrlich J., Plante T.D., Smirnov A., Shire D.B., Gingerich M., Rizzo J.F. (2009). Sputtered iridium oxide films for neural stimulation electrodes. J. Biomed. Mater. Res. - Part B Appl. Biomater..

[47] Loudin J.D., Simanovskii D.M., Vijayraghavan K., Sramek C.K., Butterwick A.F., Huie P., McLean G.Y., Palanker D.V. (2007). Optoelectronic retinal prosthesis: System design and performance. J. Neural Eng..

[48] Yang F., Chang M.-Y., Yang C.-H., Teng C.-C., Fan L.-S. (2016). Flexible, high-density microphotodiode array with integrated sputtered iridium oxide electrodes for retinal stimulation. J. Micro/Nanolithography, MEMS, MOEMS.

[49] Corna A., Herrmann T., Zeck G. (2018). Electrode-size dependent thresholds in subretinal neuroprosthetic stimulation. J. Neural Eng..

